# Extremely flat band in bilayer graphene

**DOI:** 10.1126/sciadv.aau0059

**Published:** 2018-11-09

**Authors:** D. Marchenko, D. V. Evtushinsky, E. Golias, A. Varykhalov, Th. Seyller, O. Rader

**Affiliations:** 1 Helmholtz-Zentrum Berlin für Materialien und Energie, Elektronenspeicherring BESSY II, Albert-Einstein-Straße 15, 12489 Berlin, Germany.; 2 Institut für Physik, Technische Universität Chemnitz, Reichenhainer Str. 70, 09126 Chemnitz, Germany.

## Abstract

We propose a novel mechanism of flat band formation based on the relative biasing of only one sublattice against other sublattices in a honeycomb lattice bilayer. The mechanism allows modification of the band dispersion from parabolic to “Mexican hat”–like through the formation of a flattened band. The mechanism is well applicable for bilayer graphene—both doped and undoped. By angle-resolved photoemission from bilayer graphene on SiC, we demonstrate the possibility of realizing this extremely flattened band (< 2-**meV dispersion), which extends two-dimensionally in a k-space area around the $\bar{K}$ point and results in a disk-like constant energy cut. We argue that our two-dimensional flat band model and the experimental results have the potential to contribute to achieving superconductivity of graphene- or graphite-based systems at elevated temperatures.

## Teaser

In both experiment and theory, we demonstrate a novel mechanism of flattened band formation in a honeycomb lattice bilayer.

## INTRODUCTION

Bilayer graphene (BLG) has, along with related two-dimensional (2D) materials, extensively been studied by both transport and photoemission measurements. It is a material with an energy gap that opens as soon as an asymmetry is imposed on the two graphene layers. This tunable gap framed by van Hove singularities results from the “Mexican hat” shape of the band structure (*1*) and is promising for low–power consumption transistors for which on/off ratios of 3.5 × 10^4^ are expected (*2*). By using a substrate or even sandwiching the BLG with other 2D systems, it is possible to achieve a wide range of physical phenomena related to topological properties and control them by external doping or gating: a valley Hall effect (*3*) with peaked Berry curvature at the valley bottom, a gate-tunable topological valley transport (*4*), and unconventional quantum Hall effects (*5*).

On the other hand, frequent reports of superconductivity in graphite at elevated temperatures even above 300 K (*6*) raise a number of questions. It is important to note that there is an established low- and medium-temperature superconductivity in carbon known as a phenomenon with strong doping dependence and connected to alkali and alkaline earth metals. Examples are intercalated graphite such as CaC_6_ [*T*_C_ = 11.6 K (*7*)], which can also be thinned down to an intercalated bilayer as a 2D superconductor [C_6_CaC_6_ with *T*_C_ = 4 K (*8*)], as well as doped fullerides such as Cs_2_RbC_60_ [*T*_C_ = 33 K (*9*)]. Here, the alkali and alkaline earth metals act to increase the carrier concentration and density of states (DOS) at the Fermi energy. Most recently, superconductivity was discovered in twisted BLG without any alkali doping and a *T*_C_ of 1.7 K (*10*).

The superconducting pairing mechanism is not fully established in these materials, but for many of them, there is strong evidence for phonon-mediated pairing and the validity of the Bardeen-Cooper-Schrieffer (BCS) theory (*11*). The BCS theory predicts the relation *T*_C_ ∝ exp[− 1/(*Un*(*E*_F_))] between the critical temperature *T*_C_ of superconductivity, the coupling constant *U* of the effective attractive interaction, and the DOS at the Fermi energy *n*(*E*_F_) such that, according to the BCS theory, *T*_C_ can be enhanced by increasing either *U* or *n*(*E*_F_).

The characteristic feature of graphite and graphene is, however, their low or zero density of electronic states at the Fermi level, with linear dependence on the energy. It has been argued that a flat band system will enable superconductivity with strongly enhanced *T*_C_ values (*12*). While *U* remains difficult to assess, a flat band will lead to maximal values of *n*(*E*_F_). Moreover, in this case of *U* ≫ *W*, the BCS theory predicts *T*_C_ ∝ *Un*(*E*_F_); i.e., the exponential suppression of *T*_C_ with the interaction strength is removed (*12*). In this context, rhombohedral (i.e., ABC) stacking of multilayered graphene has been considered in theory (*12*, *13*). Consequently, there is much interest to realize the rhombohedral stacking in experiment, and recently, angle-resolved photoemission spectroscopy (ARPES) has shown a flat band for five-layer graphene on 3C-SiC, which resembles the calculated very flat dispersions (*14*). In the photoemission data, the flat band is found to extend near $\bar{K}$ by about 0.8 Å^−1^ (*14*). In this range, the band disperses by only ~ 20 meV. Over the past years, theory has consistently predicted flat band superconductivity accessible by doping or gating and with enhanced critical temperature. These theoretical approaches were inspired by cases such as the ABC-stacked graphite or the van Hove singularity at the $\bar{M}$ point of monolayer graphene (MLG) and would substantially gain relevance if an extremely flat band were found (*15*–*17*). Most recently, superconductivity has been observed in twisted BLG (*10*, *18*) as the first purely carbon-based 2D superconductor. For “magic angles” between the two graphene layers, the moiré pattern leads to flat band formation (*19*), and the observed superconductivity is directly assigned to the flat band effect.

In the present study, we investigate another way of band flattening and DOS enhancement for the system with low DOS: BLG on SiC, which does not require any twist. We use high-resolution ARPES measurements to reveal the Mexican hat band structure of BLG on SiC in detail. We show that there is a band portion that is much flatter, narrower, and of higher photoemission intensity than expected, showing experimentally no dispersion around the graphene $\bar{K}$ point (for ~ ±0.017 Å^−1^). This means that a very high DOS is compressed here at a narrow energy interval. This strong peak in the graphene DOS is, first of all, promising for the application of this system in high on/off ratio graphene-based transistors (*2*). Because this extremely flattened band forms a strong 2D-extended van Hove singularity and we find indications of enhanced electron-phonon coupling, it could be used to achieve high-temperature superconductivity in BLG.

On the basis of our theoretical analysis, we propose a novel mechanism of band flattening in BLG by biasing of only one sublattice relative to other sublattices, which is possible for both doped and undoped BLG. We show that the band flattening effect is universal and achievable by different means of combining interlayer asymmetry, sublattice asymmetry, and doping. The mechanism allows control of the band dispersion all the way from parabolic through flat band formation to Mexican hat–like.

## RESULTS

### Experiment

**Fig. 1:**
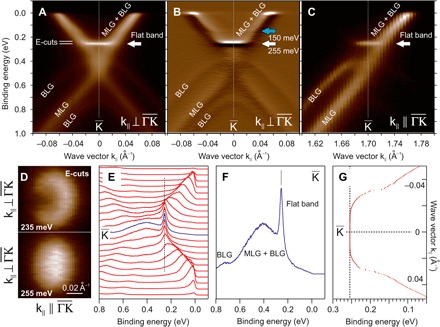
Angle-resolved photoemission spectroscopy. (**A**) Data for the sample with 1.2 monolayer graphene (MLG) coverage around the $\bar{K}$ point of the graphene Brillouin zone. The MLG Dirac cone dispersion, the faint BLG dispersion, and an intense nondispersing flattened band at 255-meV binding energy, marked by a white arrow, can be seen. Measurements were done at *h*ν = 62 eV and *T* = 60 K. (**B**) First derivative with respect to energy from the same data as in (A) where dispersions of all bands are much more visible. A blue arrow shows the possible presence of one more flat band. (**C**) Measurements in the $\bar{\Gamma K}$ direction showing a destructive interference effect for the monolayer and bilayer bands and its absence for the flat band. (**D**) Constant energy cuts taken at 235- and 255-meV binding energies. (**E**) Same data as in (A) presented as a stack of spectra (only every 10th spectrum is shown). (**F**) Spectra at the $\bar{K}$ point showing the flattened band intensity and its narrow width. (**G**) Dispersion of the maxima extracted from the spectra.

Figure 1 shows ARPES data for a 6H-SiC sample with 1.2 monolayer graphene (MLG) coverage. [As usual, the structural graphene monolayer at the interface, “zero-layer graphene” (ZLG), which is covalently bonded and acts as buffer, is not counted.] For this coverage, the MLG Dirac cone dispersion is expected to dominate with BLG contributing just a faint intensity. However, there is an additional peculiar, very intense, very sharp, and very flat band portion at 255-meV binding energy that is not present in the case of MLG on SiC (*20*). The new band is marked by white arrows in Fig. 1 (A to C). On the basis of both calculations and experimental data, we attribute this band to the bottom of one of the BLG bands. For this 1.2 monolayer coverage, the photoemission intensities of the BLG bands are about four times lower than those of the MLG bands, but at the same time, the flat band intensity is about three times higher than that of the MLG bands.

There are examples in the literature where it is possible to see this intense band in the data, but it has been ignored in discussions so far (*21*–*23*), possibly because the resolution was not sufficient for details of the band dispersion. We performed the measurements in Fig. 1A with the electron wave vector perpendicular to the $\bar{\Gamma K}$ direction and through the $\bar{K}$ point, at a temperature of 60 K and a photon energy of 62 eV. We did not observe any difference between data at $\bar{K}$ and $\bar{K\prime }$. The features are much better visible as first derivative with respect to energy in Fig. 1B: There is a faint indication that possibly one more flat band at 150 meV (blue arrow) exists together with a kink in the dispersion in the 150- to 160-meV energy range.

To judge the photoemission intensity distribution, we measured a 3D map around the $\bar{K}$ point. The cut along the $\bar{\Gamma K}$ direction in Fig. 1C reveals that, in this experimental geometry, only half of the monolayer Dirac cone and only half of the bilayer dispersion are visible because of a destructive interference from the two graphene sublattices (*24*, *25*). We see the flat band on both sides from the $\bar{K}$ point with similar intensities. This is unusual for photoemission interference from graphene. In Fig. 1D, two constant energy cuts are presented, taken from Fig. 1A data at 235- and 255-meV binding energies. An energy difference of 20 meV, very small for ARPES otherwise, is enough to show the drastic change in the constant energy cuts. At 235 meV, away from the flat band, there is a nearly circular ring with intensity modulation due to the photoemission interference effect, but at 255 meV, one sees the shape of a disk, without modulation by interference.

The representation in Fig. 1E as a stack of photoemission spectra (only every 10th spectrum is shown) demonstrates the high photoemission intensity of the flat band. Figure 1F shows the spectrum that exactly intersects the $\bar{K}$ point. The spectra were analyzed by simple single-peak Gaussian fitting of the topmost portion of the photoemission peaks. The resulting dispersion is shown as dots in Fig. 1G. We find that the scattering of peak maxima energies is not more than 2 meV for a **k**-space range of ±0.017 Å^−1^ around the $\bar{K}$ point. In addition to the flat band at 255 meV, there is a kink and a second flat band faintly visible around 150-meV binding energy (Fig. 1, B and G, and fig. S1).

The sample with mostly BLG contribution is presented in fig. S1. We measured the data at room temperature; hence, the energy broadening is slightly larger, but all the features under discussion are well visible. In summary, at the position of the bottom of one of the BLG bands, we have a nondispersive and very sharp band with more than an order of magnitude higher photoemission intensity than expected.

### Density functional theory

**Fig. 2:**
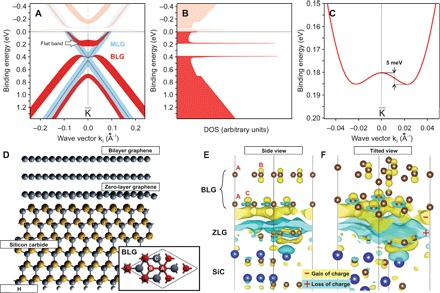
DFT calculations. (**A**) Calculated band structures of MLG (blue) and BLG (red) on SiC around the $\bar{K}$ point with *k*_∥_ perpendicular to the $\bar{\Gamma K}$ direction. Width of lines corresponds to the contribution of *p*_*z*_ orbitals to the top graphene layer. (**B**) DOS calculated from the data in (A), taking into account the wave function contribution to both layers of BLG and assuming rotational symmetry of the calculated bands around the $\bar{K}$ point. (**C**) Magnified view of the calculated flat band dispersion around the $\bar{K}$ point. (**D**) Slab structure of BLG on 6H-SiC(0001) used in DFT calculations. Between the BLG and the SiC substrate, there is a graphene buffer (zero) layer. The back side of the slab is H passivated. The inset shows the model of the BLG/6H-SiC unit cell used in the calculations (only BLG is shown), with definition of sublattices A, B, and C used in the present work. (**E**) Effect of BLG interaction with the ZLG/SiC system (ZLG/SiC). Yellow-colored isosurfaces show gain of charge, and light blue–colored isosurfaces show loss of charge, indicating charge transfer from the ZLG/SiC system to the BLG (mainly bottom layer) and emergence of a strong sublattice asymmetry. Letters A, B, and C in the top-left part of the figure indicate A, B, and C sublattices, correspondingly. (**F**) Same as (E) with tilted view for better clarity.

We conducted density functional theory (DFT) calculations of MLG, BLG, and trilayer graphene (TLG)/6H-SiC systems using the Vienna Ab initio Simulation Package (VASP) package (see overview in fig. S2) (*26*). The structure of the BLG/SiC used in the calculations is presented in Fig. 2D. To compare with experimental data, the DFT dispersions of both the MLG and BLG are presented in Fig. 2A. The experimentally observed picture and the high DOS are well reproduced. In Fig. 2C, we see a magnified view of the calculated flat band structure with a 5-meV dispersion around the $\bar{K}$ point. This value is small relative to the overall band structure but significantly larger than the flat band dispersion observed in our experiment.

Symbol sizes in Fig. 2A reflect the contribution of *p*_*z*_ orbitals in the topmost graphene layer to the calculated band structure. We see a strong localization of the flat band wave functions on the topmost graphene layer. A closer examination shows that the flat band wave function is localized not only on the top graphene layer but also on solely one graphene sublattice, B (see fig. S3). We observed a similar effect in graphene/Ni(111), where upper and lower halves of the Dirac cone belong to different sublattices (*27*). We will return to this point below since it helps us develop the low-energy Hamiltonian for the present problem.

Assuming rotational symmetry of the calculated bands around the $\bar{K}$ point, as is supported by the experimental data in Fig. 1, we calculate the DOS from the data in Fig. 2A, taking into account both layers of the BLG. The result, presented in Fig. 2B, shows two very strong DOS singularities at the edges, separated by a gap. These DOS peaks represent van Hove singularities, which, in the case of the flat band, have a very strong DOS divergence because d*E*/d*k*, d^2^*E*/d*k*^2^, and d^3^*E*/d*k*^3^ are zero at the $\bar{K}$ point and the flat band is spread in **k**-space as a 2D disk-like area in the Brillouin zone.

To investigate the role of the substrate for the flat band appearance, we calculated the charge density redistribution in the BLG due to interaction with the SiC substrate (ZLG/SiC) as a difference ρ_BLG/SiC_ − ρ_BLG_ − ρ_SiC_. Details of the charge density redistribution provide us information on the interplay between interlayer and sublattice asymmetries in BLG on SiC, important for the formation of specific band shapes different from the case of a free BLG. The result is presented in Fig. 2 (E and F). Yellow-colored isosurfaces show gain of charge, and light blue–colored isosurfaces show loss of charge, indicating both charge transfer from the ZLG/SiC system into the BLG (mainly the bottom layer) and emergence of a strong sublattice asymmetry at the bottom layer. From this picture, we can also see that both sublattices A and B of the top graphene layer and the sublattice A of the bottom graphene layer are almost unaffected and show only small charge asymmetry due to the interaction with the substrate. However, the C sublattice, which is in the lower graphene layer, is strongly affected by the interaction. We will further investigate this finding below.

Flat bands are also generally unstable against other types of order, such as ferromagnetism and charge order, and much of the argumentation in the present work holds for these cases as well. 2D ferromagnetism has been investigated theoretically in gapped BLG (*28*). Comparing the tendencies for ferromagnetism, charge order, and superconductivity in flat band systems, it was recently concluded that superconductivity will always prevail, provided the flat band is brought sufficiently close to the Fermi level (*15*–*17*). This has been concluded particularly for the flat band in ABC-stacked graphite (*15*) and for pure MLG with van Hove singularity (*15*, *17*) and is also valid for the present case.

### Model Hamiltonian

Intuitively, the simplest form of the BLG Hamiltonian around the $\bar{K}$ point can be constructed by joining two MLG 2 × 2 matrices into a larger 4 × 4 matrix and adding the required interaction terms. This results in the Hamiltonian *H*_1_ (Eq. 1), where *t* and *b* subscripts denote top and bottom graphene layers, respectively, and superscript indices indicate BLG sublattices A, B, and C (see the inset in Fig. 2D). Sublattice A atoms of the top layer are located above sublattice A atoms of the bottom layer, and in the first approximation, we add only the interaction between them (*t*_⊥_), neglecting interactions between other pairs of atoms located at larger distance from each other. We get to Hamiltonian *H*_2_ when we consider the top layer graphene atoms to be at the same energy *E*_*t*_ (i.e., without sublattice asymmetry) and the bottom graphene atoms to experience sublattice asymmetry Δ due to the interaction with the substrate. The substrate also causes a difference between *E*_*t*_ and *E*_*b*_, which represent interlayer asymmetry between top and bottom layers, due to the interaction with the substrate and the corresponding charge transfer. This Hamiltonian (Eq. 1) can also be considered a simplified version of the Slonczewski-Weiss-McClure model for bulk graphite (*29*) adapted to BLG.

$$H_{1}=(\begin{array}{cccc} E_{t}^{A} & \pi & t_{⊥} & 0\\ \pi * & E_{t}^{B} & 0 & 0\\ t_{⊥} & 0 & E_{b}^{A} & \pi \\ 0 & 0 & \pi * & E_{b}^{C} \end{array})H_{2}=(\begin{array}{cccc} E_{t} & \pi & t_{⊥} & 0\\ \pi * & E_{t} & 0 & 0\\ t_{⊥} & 0 & E_{b}-\frac{\Delta }{2} & \pi \\ 0 & 0 & \pi * & E_{b}+\frac{\Delta }{2} \end{array})$$ (1)

where $\pi =\frac{\sqrt{3}}{2}at(k_{x}+ik_{y})$, with *a* being the graphene lattice constant and *t* being the nearest-neighbor hopping energy.

Fitting the Hamiltonian parameters to reproduce the experimentally observed band structure yields values of *E*_*t*_ = 0.25 eV, *E*_*b*_ = 0.35 eV, and Δ = 0.2 eV (modeling with 0.01-eV precision). From these values, we immediately see the important property

$$E_{t}=E_{b}-\frac{\Delta }{2}$$ (2)

Returning to the more general case of Hamiltonian *H*_1_ shows that there are several possible conditions that yield a flat band (several orders of derivatives equal to zero at the $\bar{K}$ point); they differ mostly in how charges are redistributed between sublattices and with which sign. Only one case was found to reproduce a result reasonable from the point of view of charge transfer signs and magnitudes for the case of BLG on SiC

$$E_{t}^{B}=E_{b}^{A}\;\;\;\;\;E_{b}^{A}<E_{b}^{C}$$ (3)

In other words, the bottom-layer sublattice A is energetically equal to the top-layer sublattice B. This condition is possible in the case of both interlayer and sublattice asymmetries compensating each other at the A sublattice of the bottom layer. Note that, here, the $E_{t}^{A}$ value is unimportant for obtaining the flat band.

**Fig. 3:**
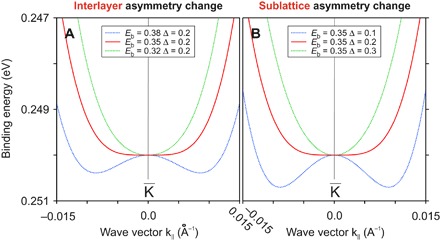
Interlayer/sublattice asymmetry. Demonstration of the possibility of transforming the flat band electronic structure into a band with either positive or negative effective mass by changing (**A**) the interlayer asymmetry and (**B**) the sublattice asymmetry of the bottom layer. The calculation is based on the Hamiltonian *H*_2_.

At the same time, any deviation from Eq. 3 by changing either the sublattice or the interlayer asymmetry values produces a band with finite curvature around the $\bar{K}$ point. Figure 3 shows this situation in which a slight change of parameters modifies the flat band structure either into a parabolic shape (positive effective mass) or into a Mexican hat shape (negative effective mass), changing in particular the number and positions of the band minima. In the intermediate stage, we have a band with the first, second, and third derivatives equal to zero at the $\bar{K}$ point. If we include more interaction parameters into the Hamiltonians (Eq. 1), the overall picture will appear distorted, but the possibility of achieving a flat band and the fine-tuning of the band shape remain unaffected.

The condition (Eq. 3) for the graphene sublattice energies is very close to the picture that we have in DFT calculations concerning the charge density redistribution due to graphene-substrate interaction (Fig. 2, E and F). As we have shown experimentally, these parameters are present in the BLG on the SiC system; however, they could be reproduced in BLG or TLG on other substrates by the joint influence of sublattice and interlayer asymmetries, e.g., by combination of chemical doping and gating. Furthermore, the proposed mechanism is promising beyond graphene because it is also applicable to other 2D layered systems such as multilayers of germanene and silicene.

## DISCUSSION

The experimental high photoemission intensity of the flat band can be partially explained by the 2D extent and broadening in *k*_*x*_ and *k*_*y*_. This broadening, however, is not the reason for the flatness and the observed features of the band. If this broadening were to play a significant role, we would see the narrowing and intensity enhancement effects also at other BLG bands around the $\bar{K}$ point where their d*E*/d*k* becomes locally zero. The experiments do not show these effects.

In Fig. 1C, we see, unusual for graphene, the disappearance of the interference pattern in the region of the flat band, resulting (Fig. 1D) in a disk-like image of the constant energy cut at 255-meV binding energy. The destructive interference arises because of localization of the wave function on different graphene sublattices (*24*, *25*); however, for the flat band, the wave function is localized on one sublattice only, and preconditions for the destructive interference disappear (fig. S3).

In the ARPES data, there are, in addition to the pronounced flat band, a kink and a second flat band, faintly visible around 150-meV binding energy (Fig. 1, B and G, and fig. S1). The nature of this kink is not unambiguous as two different reasons could produce a similar result. First, it could be due to overlap of intensities from regions with different numbers of graphene layers, particularly TLG.

Calculations of TLG on 6H-SiC in two possible stackings (ABA and ABC) are presented in fig. S2. In fig. S4, they are shown taking into account the contribution of the wave function to the top graphene layer and overlapped with the BLG for comparison. From these figures, we see that the rhombohedral (ABC) TLG on SiC has its own flat band structure with specific electron localization at a binding energy lower than that of BLG. This TLG coverage may actually be negligibly small, especially in the case of an extremely sharp and intense photoemission feature. For undoped TLG, the band structure was studied experimentally by Nanospot ARPES and shows cubic band dispersion at the Fermi level (*30*) for rhombohedral stacking. An example of MLG, BLG, and TLG on another substrate, Ir(111), is presented in fig. S5. There are characteristic double- and triple-split Dirac cones without flat bands. Because of the absence of n-doping, only the bottom bands are visible.

Another possible explanation for the observed kinks is renormalization due to many-body effects as known from MLG (*20*, *31*). The enhanced electron-phonon coupling in superconducting CaC_6_ produces in ARPES a very similar renormalization around 160 meV below the Fermi level (*32*). Thus, we want to address at this point again the relevance for superconductivity. There are various possible pairing mechanisms for intrinsic superconductivity in graphene. Besides conventional s-wave pairing (*33*), p + ip (*33*), d (*34*), d + id (*16*), and f (*16*) have also been considered for graphene. It should also be mentioned that the extra layer degree of freedom in bilayer systems leads to more possibilities in pairing. In this way, e.g., the possibility of interlayer pairing arises (*35*). Since the pairing mechanism is not established despite the strong indication for electron-phonon coupling, we want to briefly assess the role of strong electron correlation (*36*). It is possible that electron correlation contributes to the flatness of the band. For graphene, this has been predicted (*31*). We have performed model calculations to simulate complete photoemission spectra. As a result, the small broadening in the experiment at higher energies is incompatible with a significant role of electron correlation for the flat band dispersion.

Returning to the question of electron-phonon coupling, we note that the disk-like constant energy surface around the $\bar{K}$ and $\bar{K\prime }$ points of the graphene Brillouin zone favors enhanced intravalley and $\bar{K}\to \bar{K\prime }$ intervalley scattering processes when the flat band is shifted to the Fermi level. With small doping/gating of only a few milli–electron volts, the Fermi surface can be changed between circle and disk shapes, strongly affecting the number of possible scattering channels.

The measured band structure shows n-doping due to the substrate influence; therefore, the Dirac cone and the flat band in discussion are located significantly below the Fermi level. This means that it is necessary to bring the flat band to the Fermi energy to examine possible superconductivity. This is possible by doping (*21*) and gating (*37*). We recall that the possibility of doping large amounts of charge carriers to the graphene layer was realized by combined Ca intercalation and K deposition, resulting in bringing a 1D extended van Hove singularity along the $\bar{\text{MK}}$ direction from more than 1 eV above down to the Fermi level (*17*). In the present case, four times less doping but of the p-type must be accomplished. Fortunately, p-doping of BLG on SiC has been demonstrated as well (*21*). It was shown that F4-TCNQ molecules compensate n-doping of BLG on SiC and make it charge neutral (*21*).

Based on the proposed model, n-doping of only one graphene sublattice (either B or C) of initially undoped BLG leads to gap opening along with an instant flattening of the dispersion at $\bar{K}$. With increased doping, the flat band area increases, but the energy position remains fixed (fig. S6). In a device, however, single-sublattice doping is difficult to control. Thus, the main approach of modification of the band dispersion is supposed to be the gate biasing with corresponding change of interlayer asymmetry until both interlayer and sublattice asymmetries compensate each other at the A sublattice of the bottom layer. By using a double-gate device configuration (*37*), it should become possible to control both the doping and the interlayer asymmetries independently and in operando.

## CONCLUSIONS

Our high-resolution ARPES study of BLG on 6H-SiC shows that the band structure around the $\bar{K}$ point has neither the predicted parabolic nor the Mexican hat shape but stays in an intermediate stage causing flat band dispersion. The band has a number of unusual properties such as very high photoemission intensity, very high DOS without detectable dispersion and narrow width, contribution of only one graphene sublattice, and the absence of photoemission interference effects. We explain the mechanism of the flat band appearance and show that, by influencing sublattice and interlayer asymmetries, one can radically control the band shape and its properties. Indications of enhanced electron-phonon coupling, together with the discussed possibility of creating and controlling the flat band, are related to the question of high-temperature superconductivity in graphene- and graphite-based systems, while, on the other hand, the mechanism of the flattening of the dispersion is more universal and could be used in transport applications beyond BLG.

## METHODS

### Experiment

Measurements were performed using linearly polarized undulator radiation from the UE112 beamline and hemispherical analyzers of three experimental stations at BESSY II: “ARPES 1^2^” station equipped with a Scienta R8000 analyzer for initial high-resolution studies (*T* = 60 K), “ARPES 1^3^” station with a Scienta R4000 analyzer for high-resolution low-temperature studies (*T* = 1 K), and PHOENEXS station for room temperature core-level studies. Graphene on nitrogen-doped 6H-SiC(0001) was produced as described in (*38*). The substrate was etched in molecular hydrogen at a pressure of 1 bar and a temperature of 1425°C to remove polishing damage. Then, graphene was grown by annealing the sample in 1 bar of argon at a temperature of 1675°C. The sample was transferred in air into the photoemission station and subsequently annealed. Low-energy electron diffraction (LEED), x-ray photoelectron spectroscopy (XPS), photoemission electron microscopy, and ARPES from this sample showed that it is mostly one MLG with 20% contribution of BLG. After the initial study, the sample was stored in air, annealed at 250°C for 20 min, and studied again. The sample quality was the same without any significant changes in LEED, XPS, and ARPES. Subsequently, the sample was annealed several times in ultrahigh vacuum with increased temperatures in the range of 1300° to 1400°C, until parts of the surface appeared with mostly BLG. At all stages, ARPES and LEED measurements were conducted for control of the sample quality and the graphene layer thickness. All results shown here were from the same sample, and the same results were obtained from other samples grown at other times, which only differ in the bilayer/monolayer ratio.

### Theory

DFT calculations were performed using VASP (*26*) with the projector augmented-wave method in the generalized gradient approximation. Van der Waals forces were taken into account using the DFT-D2 method. The calculated system is a slab of six Si-C layers (*ABCACB* stacking), zero-layer graphene (buffer layer), and graphene layers on top (one for MLG, two for BLG, and three for TLG) (see Fig. 2D). The vacuum region between the slabs was about 27 Å. The back side was hydrogen passivated.

## Supplementary Material

http://advances.sciencemag.org/cgi/content/full/4/11/eaau0059/DC1

## SUPPLEMENTARY MATERIALS

Supplementary material for this article is available at http://advances.sciencemag.org/cgi/content/full/4/11/eaau0059/DC1 Fig. S1. BLG ARPES. Fig. S2. MLG, BLG, and TLG/SiC. Fig. S3. BLG sublattice contributions. Fig. S4. TLG/SiC. Fig. S5. Graphene/Ir thickness dependence. Fig. S6. Unsupported BLG.
