# Supplementary material for: Extremely flat band in bilayer graphene
Source: Sci Adv. 2018 Nov 9;4(11):eaau0059. doi: 10.1126/sciadv.aau0059 (PMC6226281; doi:10.1126/sciadv.aau0059)
Supplement: http://advances.sciencemag.org/cgi/content/full/4/11/eaau0059/DC1 [file supp_4_11_eaau0059__index.html]

Science Advances | Science Advances

## Supplementary Materials

**This PDF file includes:**

- Fig. S1. BLG ARPES.
- Fig. S2. MLG, BLG, and TLG/SiC.
- Fig. S3. BLG sublattice contributions.
- Fig. S4. TLG/SiC.
- Fig. S5. Graphene/Ir thickness dependence.
- Fig. S6. Unsupported BLG.

Download PDF

**Files in this Data Supplement:**

- Adobe PDF - aau0059\_SM.pdf
