# Supplementary material for: Extremely flat band in bilayer graphene
Source: Sci Adv. 2018 Nov 9;4(11):eaau0059. doi: 10.1126/sciadv.aau0059 (PMC6226281; doi:10.1126/sciadv.aau0059)
Supplement: http://advances.sciencemag.org/cgi/content/full/4/11/eaau0059/DC1 [file aau0059_SM.pdf]

Science Advances
AAAS

advances.sciencemag.org/cgi/content/full/4/11/eaau0059/DC1

Supplementary Materials for

Extremely flat band in bilayer graphene

D. Marchenko*, D. V. Evtushinsky, E. Golias, A. Varykhalov, Th. Seyller, O. Rader

*Corresponding author. Email: marchenko.dmitry@gmail.com

Published 9 November 2018, *Sci. Adv.* **4**, eaau0059 (2018)
DOI: 10.1126/sciadv.aau0059

This PDF file includes:

Fig. S1. BLG ARPES.
Fig. S2. MLG, BLG, and TLG/SiC.
Fig. S3. BLG sublattice contributions.
Fig. S4. TLG/SiC.
Fig. S5. Graphene/Ir thickness dependence.
Fig. S6. Unsupported BLG.

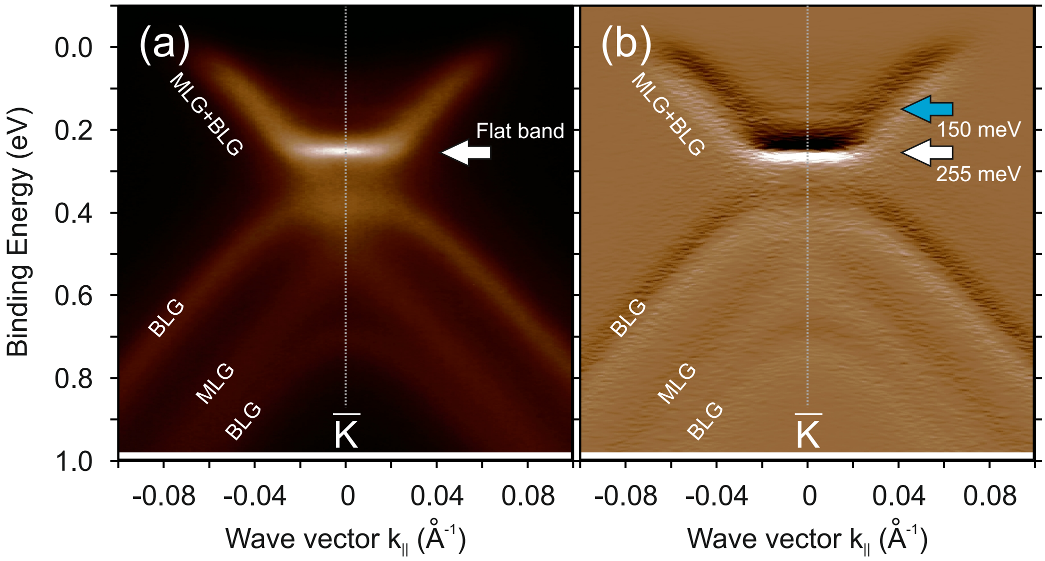

Fig. S1. BLG ARPES. (**a**) Data for the sample with mostly bilayer graphene coverage around the K point of the graphene Brillouin zone in perpendicular to ΓK direction. There is also a faint presence of the monolayer graphene Dirac cone dispersion and intense nondispersing flat band at 255 meV binding energy, marked by a white arrow. Measurements were done at h*ν*=40 eV and room temperature. (**b**) First derivative over energy from the same data as in (a) where dispersions of all bands are much better visible. A blue arrow around 150 meV shows possible presence of one more flat band, presumably from the trilayer graphene inclusions.

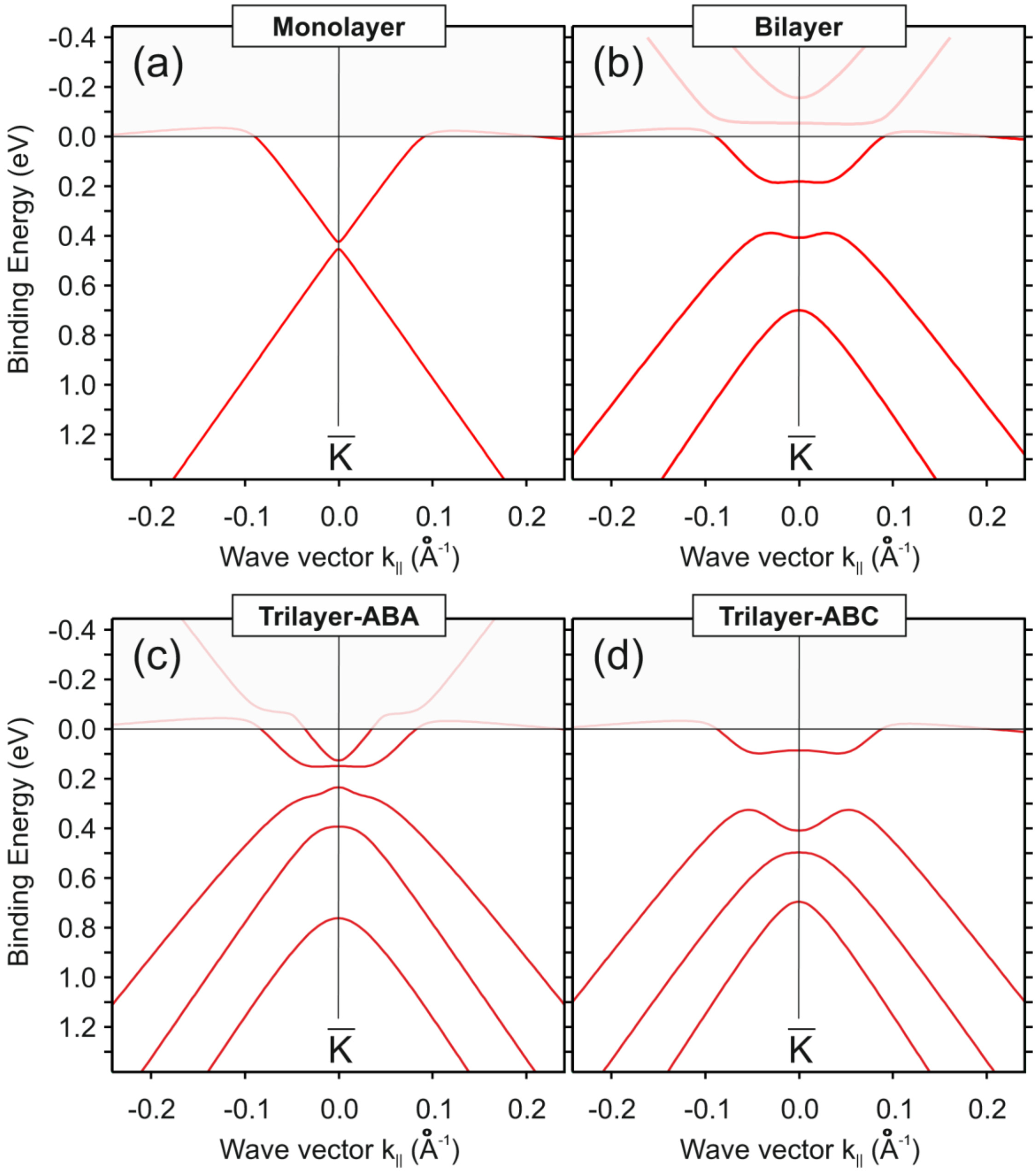

Fig. S2. MLG, BLG, and TLG/SiC. DFT calculated electronic structure of graphene on 6H-SiC(0001): (**a**) monolayer (MLG), (**b**) bilayer (BLG), (**c**) trilayer (TLG) with ABA-stacking and (**d**) trilayer with ABC-stacking.

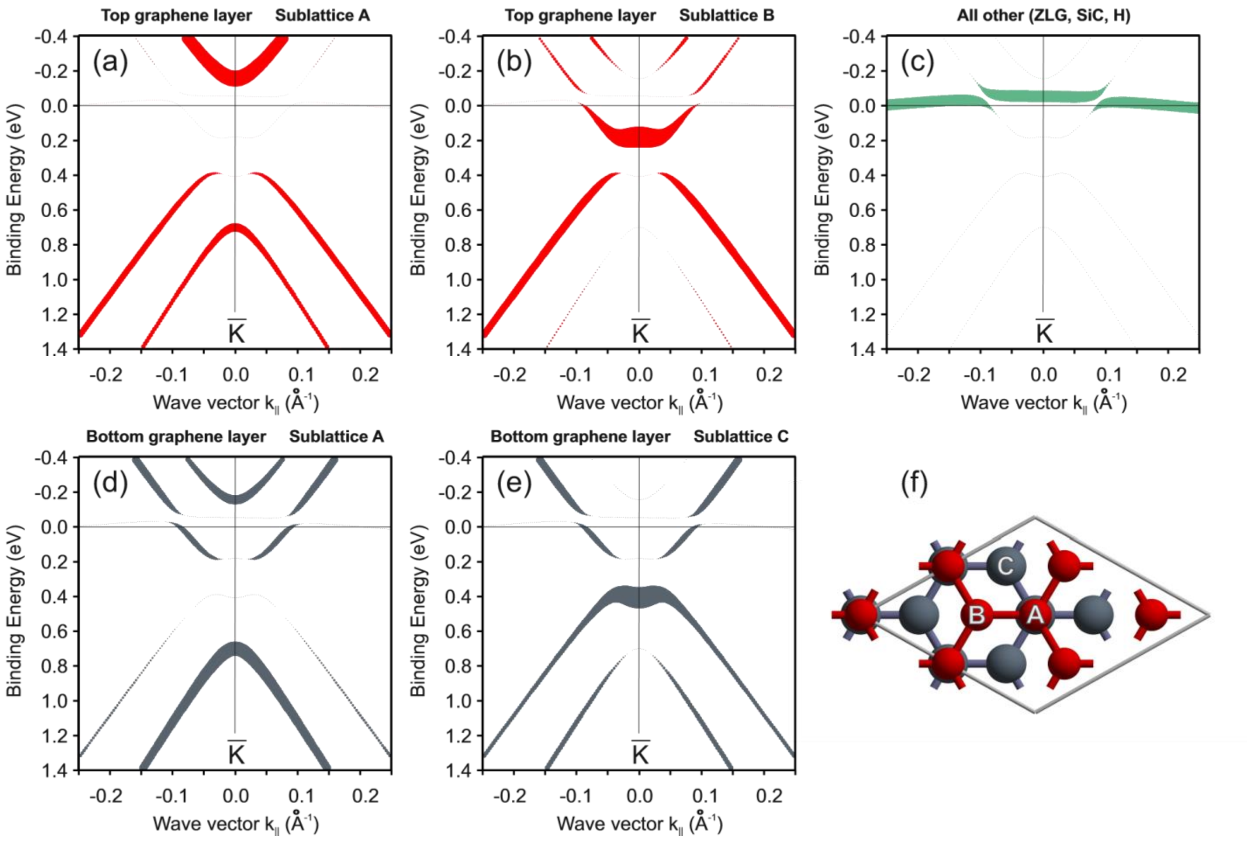

Fig. S3. BLG sublattice contributions. Bilayer graphene on SiC. (**a**, **b**) Line width shows wavefunction contribution to the top graphene layer sublattices A and B. (**c**) Contribution to all layers below the bilayer graphene (ZLG+SiC+H). (**d**, **e**) Contribution to the bottom layer of the bilayer graphene (sublattices A and C). (**f**) Model of the BLG/6H-SiC unit cell used in the calculations (only bilayer graphene is shown) with definition of sublattices A, B and C used in the present work.

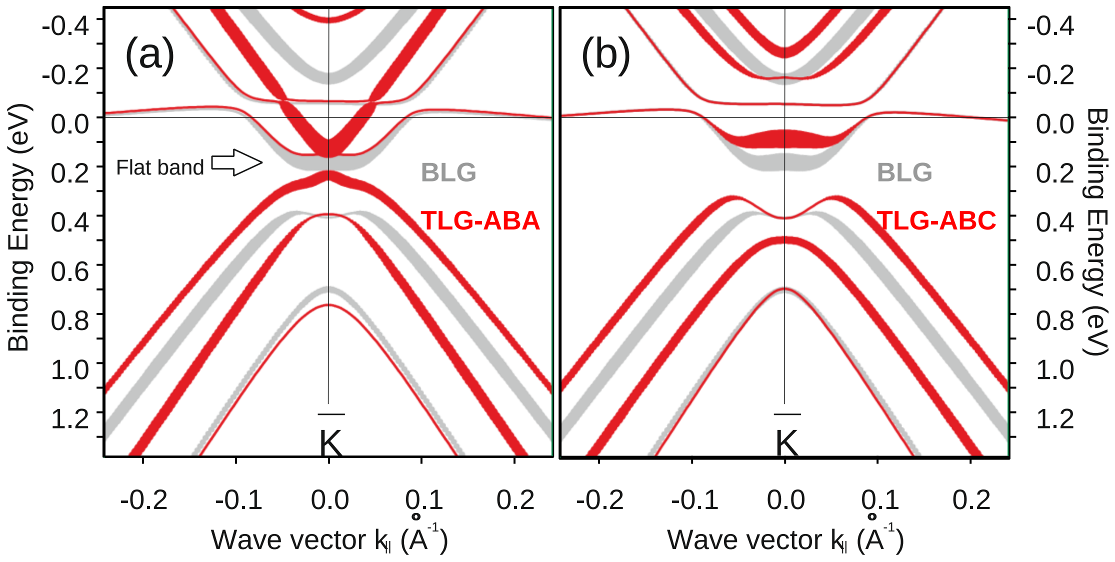

Fig. S4. TLG/SiC. Red lines show a DFT calculation result for trilayer graphene on 6H-SiC(0001) in two stacking versions: (**a**) ABA stacking, (**b**) ABC stacking. Line width shows contribution of the wavefunction to the topmost graphene layer. Grey dispersions show bilayer graphene results for comparison.

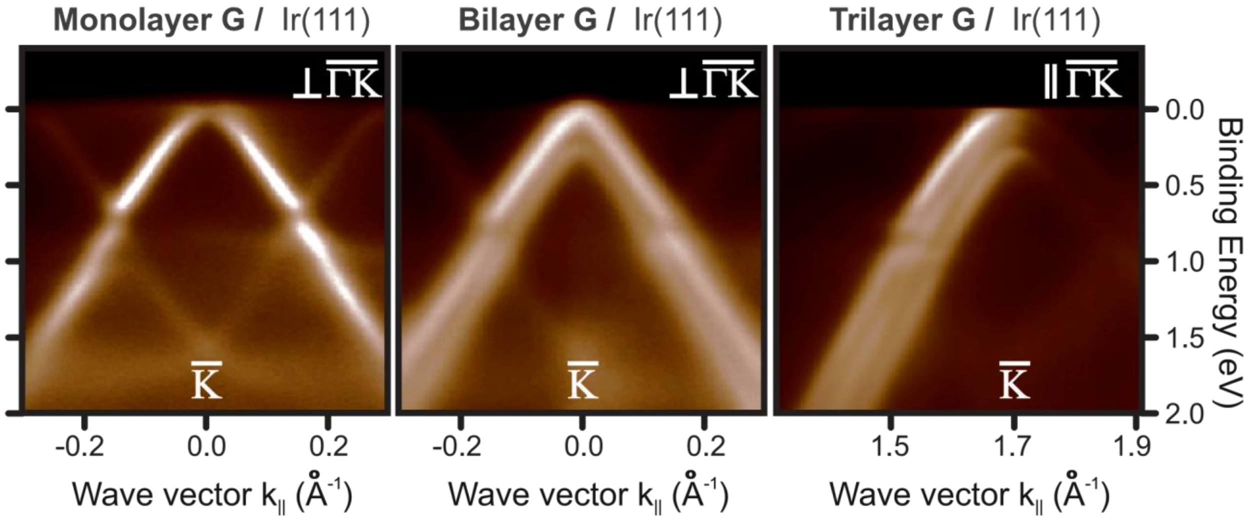

Fig. S5. Graphene/Ir thickness dependence. ARPES data around the K point of the graphene Brillouin zone for the Dirac cone bandstructure of monolayer, bilayer and trilayer graphene on iridium. These data were published in J. Sánchez-Barriga et. al., New J. Phys. **15**, 115009 (2013).

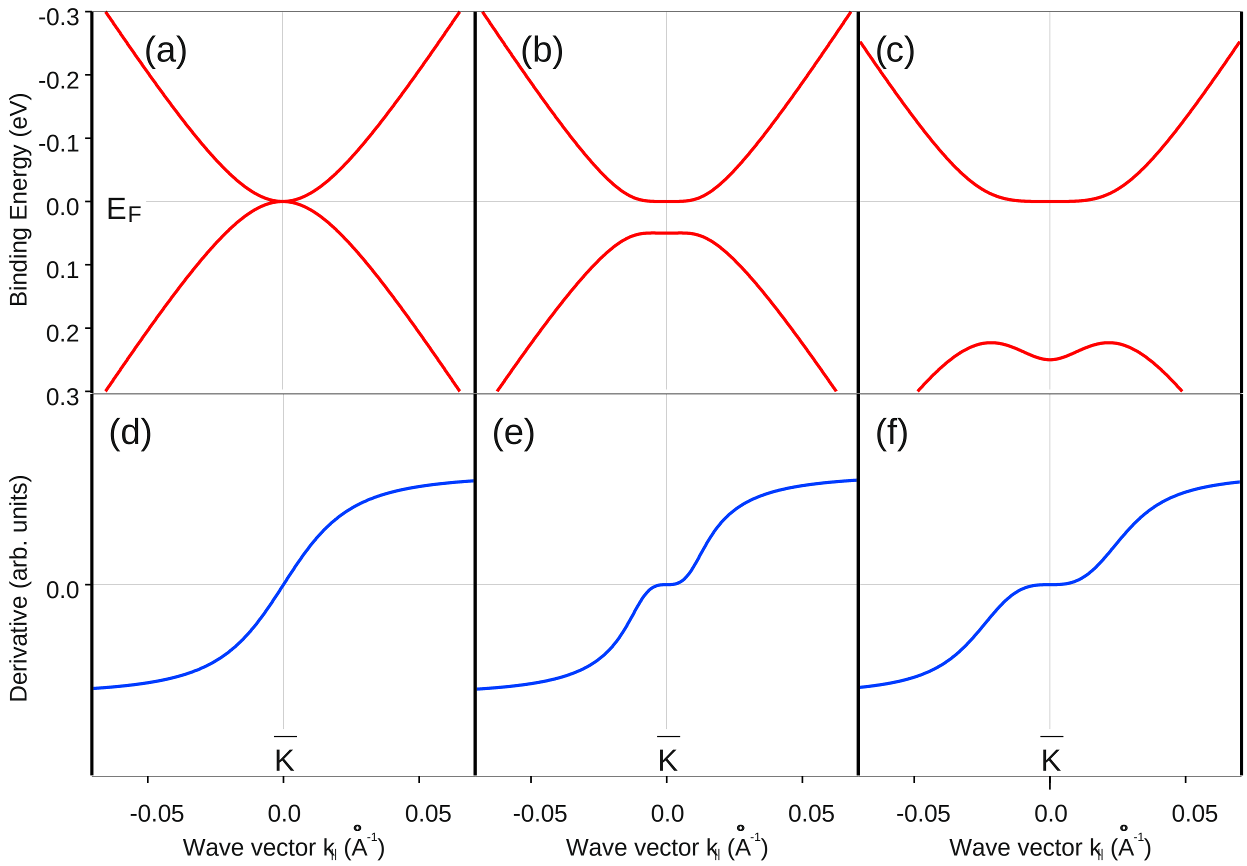

Fig. S6. Unsupported BLG. Model calculations for the freestanding bilayer graphene with n-doping of only one graphene sublattice (either B or C). (**a-c**) bandstructure around K and E_F, (**d-f**) the first derivative of the upper band of the corresponding dispersions. For such one-sublattice doping, the gap opening comes along with an instant flattening of the dispersion at K. With increased doping the flat band area increases but the energy position remains fixed.
